# Plants under pressure: the impact of environmental change on plant ecology and evolution

**DOI:** 10.1186/s12862-023-02115-z

**Published:** 2023-04-20

**Authors:** Isabel C. Barrio, Alessandro Rapini

**Affiliations:** 1 Faculty of Environmental and Forest Sciences, Agricultural University of Iceland, Reykjavík, Iceland; 2 Department of Biological Sciences, The State University of Feira de Santana, Feira de Santana, Brazil

## Abstract

Plants have demonstrated tremendous resilience through past mass extinction events. However, anthropogenic pressures are rapidly threatening plant survival. To develop our understanding of the impact of environmental change on plant ecology and evolution and help solve the current biodiversity crisis, *BMC Ecology and Evolution* has launched a new article Collection titled “Plants under Pressure”.

Plants have played a critical role in shaping the environment. For instance, the spread of forests in the Palaeozoic era, 541-252 million years ago (mya), increased rock weathering and organic carbon burial, which reduced atmospheric CO_2_ levels. This drop in CO_2_ led to global cooling and boosted shallow sea eutrophication, causing episodes of marine anoxia and extinctions [1]. However, how plants, in turn, respond to changing environments needs to be further explored.

Analyses of the fossil record suggest that plants have generally been much better at surviving, recovering, and adapting through mass extinction events than animals, and used these previous mass extinction events as an opportunity to diversify. This apparent resilience to global change is because plants possess mechanisms that enable them to resist environmental stresses and colonise new sites. For example, long-lived plant propagules can remain dormant until conditions improve for growth. Furthermore, plants can efficiently reproduce asexually, rapidly colonising barren substrates.

However, plants are not immune to ecological shifts. For instance, the mass extinction event at the end of the Cretaceous (66 mya) significantly altered the structure and composition of rainforests. While some plant groups, such as the gymnosperms, suffered, other groups, such as the angiosperms, experienced a rapid increase in their diversity, paving the way for the evolution of neotropical rainforests found today [2]. Since the Eocene–Oligocene climatic transition (34 mya), the distribution of rainforests has shrunk to lower latitudes in parallel with Cenozoic long-term cooling and aridification. More recently, in the last 50,000 years, rapid warming following the Last Glacial Maximum and preceding the Holocene drove substantial changes in vegetation at higher latitudes, from a relatively homogenous cold-adapted steppe-tundra to a more diverse mosaic of herbaceous and woody plant communities [3].

In recent years, human actions have caused rapid and unprecedented environmental changes. Humans are pumping the fossil carbon stocked by plants for tens of millions of years into the atmosphere and transforming the carbon-sink aboveground plant biomass into a carbon source. As a result, forest degradation and the emission of greenhouse gases have tremendously impacted carbon and water cycles. Anthropogenic impacts are already several orders of magnitude higher than those caused by natural processes, putting plants increasingly under pressure and testing their resilient nature [4].

Some biomes, like the lowland Amazonian Forest, are now on the verge of no return [5]. Contemporary plant extinction rates are 500 times higher than before the Anthropocene. Worryingly, these estimates are likely to be conservative because of the lack of a comprehensive and accurate record of plant extinctions. Furthermore, the extinction lag times of plants can be extended as plant species can persist for long periods before their inevitable disappearance. Thus, many species are doomed to disappear, several unnoticed, in the coming decades, and extinction threatens an estimated 39% of known vascular plant species [6]. However, patterns of plant extinction are complex: they are not geographically uniform and their drivers can also vary over time [7]. Plant extinction rates are more pronounced in areas with high biodiversity and high levels of endemism. Thus, increased research efforts in biodiversity hotspots undergoing extensive habitat change will shed some light on global drivers and patterns of recent and future plant extinctions.

From the tropics to the Arctic, threats to plants include a variety of stressors, from land use to environmental change, that affect plants directly or indirectly through their interactions with other organisms. The synergistic impact caused by the combination of habitat loss, overconsumption, climate change, pollution and invasive species has put plants under a level of pressure never seen before in the whole story of this successful lineage, probably surpassing the capacity of plants to adapt to the new conditions or disperse to suitable areas.

Given the increasing pace of environmental change, it is crucial to understand how phenotypic plasticity, genetic diversity and adaptation interact with ecological processes of dispersal and migration across species' ranges [8]. Also, we need to know whether plants' responses are species-specific or phylogenetically clustered, i.e. lineage-dependent, and how the genotype can affect the adaptation of endangered or ecologically functional species and crops under rapid environmental changes. Disentangling how direct and indirect effects combine to influence responses in a changing world is thus one of the key challenges at the interface of evolutionary biology, community ecology and conservation.

Recent technical and methodological advances can facilitate studying the responses of plants to these complex pressures. For instance, novel spaceborne sensors, including imaging spectroscopy, allow continuous and repeated monitoring and can enable tracking of the rapid and extensive changes in plant species distribution and community composition across broad regions [9]. These studies are complemented by detailed field studies aimed at understanding the mechanisms behind the responses of plants to ongoing environmental pressures. Increasingly, coordinated research efforts and globally distributed experiments are being used to address ecological questions at large spatial scales.

The development of standardised protocols for data collection and the use of common study designs can enable comparisons across sites and facilitate the synthesis of results at biome-wide scales. An excellent example of such initiatives is the International Tundra Experiment (ITEX; [10]), which, for more than 30 years now, has used a coordinated experimental set-up across a network of alpine and Arctic sites to investigate the responses of tundra vegetation to ongoing warming. Finally, other sources of information, such as analyses of environmental DNA in sediment records [3], can provide an invaluable resource to better understand the effects of past environmental changes and long-term ecological dynamics. Combining these tools and novel multidisciplinary approaches can move the field forward and help find solutions to the global biodiversity crisis.

Throughout their long history, plants have demonstrated a tremendous ability to survive and adapt during environmental change, but human actions are now challenging their resilience. To highlight this issue and support the United Nations’ Sustainable Development Goals (SDGs) 6: Clean water and sanitation, 13: Climate action, 14: Life below water and 15: Life on land, *BMC Ecology and Evolution* has launched a new Collection titled “Plants under Pressure”. The Collection welcomes research from around the globe on the impact of environmental change on plant ecology and evolution, threats to plant survival, halting and reversing plant biodiversity loss, and methods to monitor changes in plant species composition.

## Data Availability

Not applicable.

## Abbreviations

ITEX: International Tundra Experiment
mya: Million years ago
SDGs: Sustainable Development Goals
